# Melatonin—A Potent Therapeutic for Stroke and Stroke-Related Dementia

**DOI:** 10.3390/antiox9080672

**Published:** 2020-07-28

**Authors:** Nadia Sadanandan, Blaise Cozene, Justin Cho, You Jeong Park, Madeline Saft, Bella Gonzales-Portillo, Cesar V. Borlongan

**Affiliations:** Department of Neurosurgery and Brain Repair, University of South Florida Morsani College of Medicine, Tampa, FL 33612, USA; nas146@georgetown.edu (N.S.); bcozene@tulane.edu (B.C.); justincho@usf.edu (J.C.); youjeongpark@usf.edu (Y.J.P.); saftmad@umich.edu (M.S.); bellagonzales-portillo2024@u.northwestern.edu (B.J.-P.)

**Keywords:** antioxidant, melatonin, cerebral ischemia, oxidative stress, stem cells

## Abstract

Secreted by the pineal gland to regulate the circadian rhythm, melatonin is a powerful antioxidant that has been used to combat oxidative stress in the central nervous system. Melatonin-based therapies have been shown to provide neuroprotective effects in the setting of ischemic stroke by mitigating neuroinflammation and accelerating brain tissue restoration. Melatonin treatment includes injection of exogenous melatonin, pineal gland grafting and melatonin-mediated stem cell therapy. This review will discuss the current preclinical and clinical studies investigating melatonin-based therapeutics to treat stroke.

## 1. Introduction

Melatonin (N-acetyl-5-methoxytryptamine) is secreted by the pineal gland in the brain to regulate the circadian rhythm in mammals including light-dark cycles. The hormone has been found in other parts of the body including bile [1], cerebrospinal fluid [2], anterior chamber of the eye [3] and ovarian follicular fluid [4], suggesting a role outside of regulating the internal clock. Within the last two decades, melatonin has been recognized as an effective free radical scavenger and antioxidant. The central nervous system (CNS) is susceptible to oxidative stress. First, cerebral metabolism is highly active and the brain interacts with a majority of the oxygen entering the body [5]. Second, the CNS has limited free-radical scavengers due to the blood-brain barrier [5]. Lastly, the brain has high concentrations of iron, vitamin C and polyunsaturated fatty acids which are prone to oxidation [5]. Exogenous factors that can contribute to an excessive production of free radicals include exposure to ionizing or ultraviolet radiation, ischemia followed by reperfusion and physical or psychological stress [6]. Antioxidants like melatonin may help alleviate some of the oxidative-induced stresses caused by free radicals in the CNS.

Neuroprotection via melatonin has been explored for the treatment of stroke, which is characterized by aberrant inflammation and excessive production of free radicals. In this review, we will discuss preclinical and novel clinical studies investigating melatonin-based therapies to rescue the CNS following ischemia.

## 2. Mechanisms Behind Melatonin-Induced Neuroprotection in Experimental Stroke Models

Melatonin is effective as a free radical scavenger and an indirect antioxidant and serves as an accepted mechanism for neuroprotection. Melatonin can scavenge hydroxyl radicals (via the Fenton reaction from hydrogen peroxide) and peroxynitrite anions [7,8]. Also, it reduces lipid peroxidation in the brain that is produced by intoxication of free radical generating agents and blocks oxygen-induced toxicity [9]. From these studies, melatonin demonstrates its ability to protect neural tissue from free toxicity and has positive effects following ischemia in experimental models. 

The use of animal stroke models has contributed to the use of melatonin as a potential therapeutic. Stroke can be induced in experimental rats that have had their pineal gland removed because it is known to cause a reduction in the circulating levels of melatonin [10]. Mice that are treated with melatonin (5 mg/kg) at the beginning of reperfusion demonstrate a decrease in the gray and white matter in the brain [11]. Also, melatonin has proven to decrease the inflammatory response, blood-brain barrier permeability and cerebral edema formation in treated stroke animals. Treatment by intraperitoneal injection of melatonin (5 or 15 mg/kg in 1 mL saline) given a half hour before middle cerebral artery occlusion (MCAO) results in a decrease in the infarction volume compared to a lower or higher dose [12]. Additionally, melatonin serves as a key neuroprotective agent with functional effects due to the fact that melatonin receptor type 1A (MT1) increases amniotic epithelial cell proliferation through melatonin stimulation in experimental models [13]. 

Studies both in vitro and in vivo suggest that melatonin serves as a protector for glial cells and leads to a functional recovery, reduced inflammatory response and improved behavioral outcomes in ischemic animals. However, damage is still present in the lateral aspect of the striatum and suggests that normalization of motor protection may only require protection of the cortex [14]. Another study proved that melatonin protects against secondary cell death but not the functional deficits with interruption of cerebral blood flow since no behavioral protection was observed with treatment before the arterial occlusion. Replication of this study in vitro demonstrated similar cellular response to in vivo studies indicated by the survival of melatonin-treated astrocytes after serum deprivation or toxin exposure, 3-NP and sodium nitroprusside [15]. Nonetheless, other studies still confirm that pretreatment is proven to reduce cerebral infarction volume. 

Further studies are needed to explore alterations in glial cells that accompany melatonin treatment. Evidence suggests that improved glial cell survival after melatonin treatment protects injured neurons, since these cells secrete trophic factors [16]. Additionally, glial cells can maintain neuronal cell membrane homeostasis and aid in the cells’ enveloping action by siphoning off excess potassium or improving water handling capacity along with serving as a cystine/glutamate antiporter [17]. The beneficial effects of glial cells’ trophic factors in maintaining homeostasis and anti-glutamate toxicity make these cells effective neuroprotectors and melatonin’s protective effects aid glial cells against ischemic brain injury. 

## 3. The Use of Pineal Gland Grafts in Melatonin-Based Stroke Therapies 

Recent studies have provided evidence that cell replacement therapy (i.e., intracerebral transplantation) may be effective to alleviate a stroke [18]. The first clinical trial of a neural transplantation therapy for stroke was performed in 1998 using human-derived cells that were transplanted near the ischemic area of stroke to allow the transplanted cells to grow and replace the existing damaged brain cells [19]. Further research has expanded on this notion and focused on intracerebral transplantation of pineal gland grafts and their use in melatonin-based therapies. Using an acute stroke model, one study showcased that rats receiving rat-derived pineal gland allografts demonstrated ameliorated motor skills and a decrease in infarction volume. This enhanced neuroprotection is associated with an increase in melatonin levels in the cerebrospinal fluid and similar studies demonstrated positive results where melatonin was administered [20]. 

However, several comparable studies utilized a sample of rats that underwent a pinealectomy alongside the allograft transplantation and this particular group of rats did not receive any additional neuroprotection [21]. The transplantation alongside the host pineal gland resulted in elevated melatonin levels, which proved more effective in neuroprotection. On the other hand, a pinealectomy decreased the levels of melatonin and did not provide effective neuroprotection [22]. This suggests that pineal gland grafts are more effective because the grafts provide a steady stream of melatonin as opposed to sporadic increases in levels with exogenous delivered melatonin. Future melatonin- based stroke therapies are leading towards a dynamic level of melatonin for optimal neuroprotection over the course of stroke progression. 

The two different treatment methods, intracerebral pineal gland grafting and exogenous melatonin treatments, differ in the targeted line of cells. Experimental evidence demonstrates that pineal gland grafts reduce ischemic tissue volume 2 to 3 days after the onset of stroke as opposed to on the first day and suggests that pineal gland grafts target secondary cell death. In comparison of both treatment plans, a pineal gland graft is very invasive and entails an extensive transplantation surgery, while the exogenous melatonin treatment is less invasive. Although, it is important to consider that a pineal gland graft may provide greater ongoing treatment with chronic stroke patients because of the massive cell death that occurs after a stroke takes place [22]. Exogenous delivered melatonin may be a more effective treatment option for early acute stroke patients because it is less invasive and provides temporary relief [23]. A combination of two regimes is also being investigated as a more efficacious combined therapy. 

A potential side effect of pineal gland graft therapy to consider is graft rejection. Therefore, pineal gland graft therapies would need to be accompanied by immunosuppressive agents to ensure long-term graft survival [24]. Melatonin is proven to compliment immunosuppressive agents and is effective against viral [25] and bacterial [26] infections to provide relief upon a weakened immune system. Additionally, melatonin can serve as an immunosuppressive agent and large doses greater than 100 mg/kg decrease the antibody production in the body [27]. 

Further research is required to determine the efficacy of the pineal gland grafts and their relation to melatonin. A potential research option is to explore melatonin antagonist molecules in conjunction with a graft transplant to investigate if melatonin is providing all of the neuroprotection or another contributor such as various growth factors [28]. This could be carried out by using an antibody that specifically targets growth factors and then investigating if neuroprotection is altered upon transplantation.

## 4. The Role of Melatonin Receptors in Stem Cell Therapeutics under Stroke Conditions 

Recent advances in stem cell therapy research have highlighted the involvement of melatonin receptors in stem cell mechanisms [13]. Melatonin receptor 1 (MT1) and melatonin receptor 2 (MT2) are expressed in stem cells and are acted upon by melatonin [29,30]. It seems that melatonin’s neuroprotective effects are primarily due to the attenuation of oxidative stress and inhibition of apoptosis in both ischemic and hemorrhagic stroke [31]. An in vitro study examining the processes behind neural differentiation in amniotic epithelial cells (AEC) under stroke conditions engendered five observations — 1. Only MT1 is expressed in AEC cells not MT2 [13]. Therefore, the differential fate of AECs can be potentially modulated using the MT1 receptor. Notably, an additional investigation demonstrated that neural stem cells express MT1 [30], further accentuating melatonin’s pleiotropic capabilities in neuronal development. 2. The findings indicated that blocking MT1 abolished neuroprotection in the AECs. Conversely, the same results did not arise with the inhibition of MT2 [13]. 3. Melatonin bolstered differentiation and proliferation in AEC cells expressing MT1, mirroring previous findings [32]. Although several experiments have displayed the neuroprotective effects of exogenous melatonin therapy [33,34,35], incorporating both melatonin and stem-cells in treatment may be even more therapeutically potent [13,21]. 4. Along with melatonin’s enhancement of proliferation and differentiation in AEC cells, melatonin coupled with AEC therapy in oxidative stress disorders should also ameliorate neurodegeneration by activating anti-oxidative mechanisms [34,36,37,38]. Moreover, utilizing melatonin and AECs in conjunction demonstrates greater therapeutic benefit than just melatonin or AECs alone. 5. The findings suggested that AEC-melatonin treatment imparted neuroprotection through the release of neurotrophic elements. In peripheral regions, melatonin is involved with vascular endothelial growth factor (VEGF) [39] and in cerebellar neurons, melatonin is involved with brain-derived neurotrophic factor (BDNF) [40]. Knowledge behind melatonin’s cellular mechanisms has grown due to higher levels of VEGF observed in AECs and additional evidence demonstrating VEGF’s interactions with MT1 [13] and BDNF with MT2 [40]. Indeed, the crosstalk among melatonin receptors and neurons have been depicted in other investigations [21,40,41,42,43]. However, this particular study involving MT1 indicates that the combined use of AEC and melatonin spurs enhanced neuroprotection, as neuronal proliferation, differentiation and release of trophic factors are increased via MT1 mechanisms. Further exploration of this combined therapy using in vivo stroke models is warranted. Notably, a new sector for melatonin receptor research may involve Ramelteon, a melatonin receptor agonist, which demonstrates greater therapeutic benefits than melatonin with respect to plasma half-life, MT selectivity and high affinity [44,45].

## 5. Stroke-Induced Dementia as a Potential Target for Melatonin-Based Therapeutics

Vascular dementia and cognitive decline are two common detrimental effects that follow stroke [46]. Post-stroke cognitive impairment can range from mild cognitive decline to dementia [47]. Cerebral ischemia induces free radical formation, glutamate stress, oxidative stress and hypoxic stress, all of which contribute to neuronal injury and cognitive impairments [48]. Stroke-related dementia is associated with fluctuations in C-reactive protein (CRP), IL-6 and IL-10 in blood serum and cerebrospinal fluid [47]. The development of dementia after stroke can be associated with an increase in the vascular accumulation of Aβ, also known as cerebral amyloid angiopathy (CAA). Deposition of Aβ and CAA are exacerbated by stroke-induced damage to the perivascular region, neuroinflammation, blood-brain barrier (BBB) impairment and hypoxia [49]. In addition, murine models have indicated several biochemical markers such as cyclic AMP, response element-binding protein (CREB) and BDNF, that become dysregulated as a result of cerebral ischemia. Following ischemic injury and limited neuronal plasticity, BDNF and CREB concentrations are decreased in the hippocampus [48]. CREB indirectly influences memory via regulation of BDNF expression. BDNF is directly involved in spatial learning and activity dependent synaptic plasticity [50]. Increasing respective concentrations of CREB and BDNF in the hippocampal region alleviates cognitive impairments and memory problems [51]. Augmented BDNF levels are linked with higher rates of neurogenesis and improved cognitive function [52]. Notably, BDNF efficacy increases in ischemic sites when stem cells are administered and may further decrease infarct volume, prevent secondary cell death and provide functional recovery [53,54,55]. Due to their differentiation ability and endocrine function, stem cells are a plausible therapy to alleviate ischemia-induced cognitive impairments by targeting CREB and BDNF concentrations [56]. Melatonin receptors are involved with stem cell fate, indicating the therapeutic potential of melatonin within ischemic-associated injuries.

## 6. Novel Evidence Supporting Melatonin as an Effective Therapeutic Agent in Stroke

An overwhelming amount of recent evidence points to melatonin’s neuroprotective effects in stroke, specifically highlighting its anti-oxidative, anti-inflammatory and anti-apoptotic capabilities, as shown in Table 1. Importantly, melatonin can cross the blood brain barrier and attenuate neuronal cell death [57]. When melatonin was administered intraperitoneally to MCAO models, the brain infarct size decreased and cognitive performance was alleviated. In addition, pro-inflammatory factors dwindled and anti-inflammatory elements escalated in the stroke-affected brain. In vitro, melatonin hindered the pro-inflammatory state of microglial cells under OGD and imparted neuroprotection [58]. Additionally, melatonin administration to SH SY5Y cells under OGD/R conditions increased cell survival and diminished TNF-α, inducible nitric oxide synthase (iNOS) and nitric oxide (NO). ROS, MDA and 4-hydroxynonenal (4-HNE) were also decreased. Notably, melatonin inhibited apoptosis via Akt signaling and attenuated autophagy in the OGD culture [59]. In another study, the ability of melatonin to ameliorate apoptosis and oxidative stress spurred by irradiation in Wistar rat brainstems was examined. The rats pre-treated with melatonin demonstrated a decrease in malondialdehyde (MDA), nitric oxide (NO) and caspase-3 protein expression, along with an elevation of antioxidant enzymatic activity [60]. 

Evidently, melatonin’s therapeutic benefits in post-stroke injury may also be due to its ability to alter microglial activity and ameliorate mitochondrial dysfunction. Melatonin can transform microglia into their advantageous form in the ischemic brain through melatonin receptors. In vitro, melatonin escalated NeuN, BDNF and MAP2 and attenuated GFAP, Iba1 and caspase-3 protein under hypoxic conditions [61]. Another in vitro study demonstrated that ITH12674, a melatonin-sulforaphane hybrid, diminishes inflammatory factors in glial cells and hippocampal cultures post lipopolysaccharides (LPS) conditioning [62]. Moreover, melatonin may ameliorate post-stroke secondary injury by altering microglial activity and imparting protection against neuroinflammation [61]. In addition, melatonin can alleviate mitochondrial impairment induced by stroke. Through an elevation of OPA1 expression, melatonin ameliorated mitochondrial fusion spurred by IR injury. The abolition of OPA1 reversed the therapeutic influence of melatonin with respect to stroke-related mitochondrial dysfunction. Moreover, through the Yap-Hippo pathway, melatonin regulates OPA1- related mitochondrial fusion, attenuating reperfusion in the brain [63]. 

Although past research has elucidated that pineal gland grafts may be therapeutically effective in stroke due to the steady stream of melatonin it provides, current investigations into the efficacy of pineal gland transplantations is lacking. Nonetheless, recent studies examining the effects of pinealectomy in the brain have been conducted. For instance, pinealectomized rats demonstrated significant melatonin scarcity, lower levels of superoxide dismutase (SOD) and elevated lipid peroxidation (LPO). Endurance training ameliorated oxidative stress at certain time points but did not rehabilitate the lack of melatonin [64]. Pinealectomy spurred drastic oxidative stress in fetal eye tissues, generating an escalation in caspase-induced apoptotic pathways. The delivery of melatonin ameliorated cell death and enhanced the expression of antioxidant enzymes, along with attenuating lipid peroxidation [65]. Furthermore, melatonin serves as a crucial therapeutic agent under oxidative deprivation and pineal gland grafts may provide a long-term supply of melatonin in the ischemic brain. Despite the insufficiency of recent studies exploring pineal gland grafts, the transplantation of stem cells preconditioned with melatonin in models of neurodegenerative disease have been investigated. bone marrow mesenchymal stem cells (BMSCs) conditioned with melatonin (MT-BMSCs) were transplanted into Wistar rats. Compared to normal BMSCs, MT-BMSCs more effectively improved the cognitive function of Wistar rats [66]. Moreover, an abundance of evidence displays the neuroprotective actions of melatonin but further examination of pineal gland graft efficacy in the ischemic brain is warranted. 

**Figure 1:**
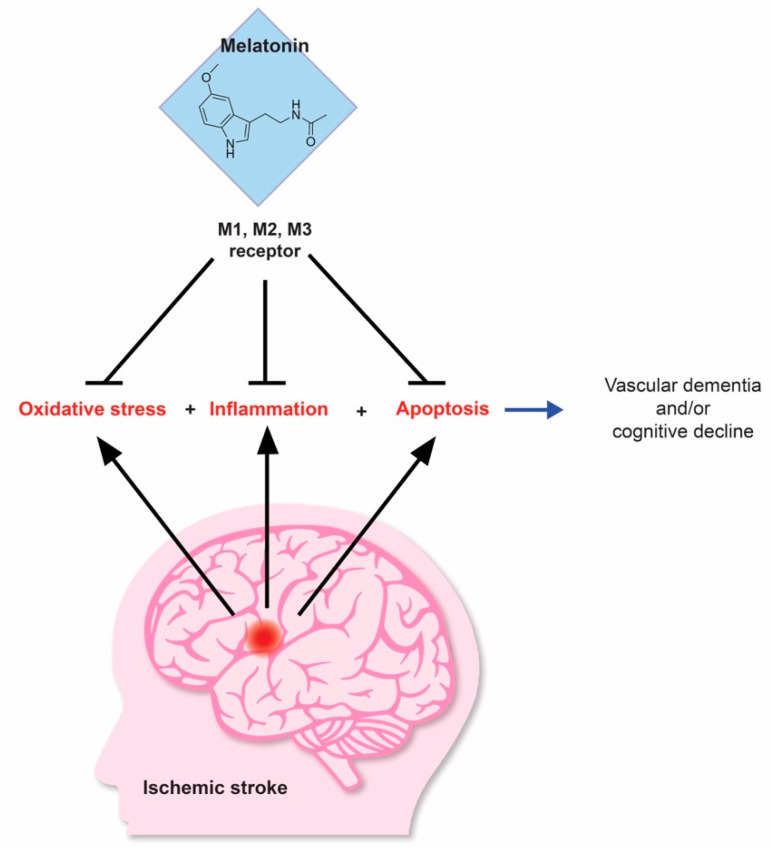
Melatonin’s Neuroprotective Effects. Through the M1, M2 and M3 receptors, Melatonin imparts therapeutic benefits combatting oxidative stress, neuroinflammation and apoptosis induced by ischemic stroke. These neuroprotective traits may also ameliorate vascular dementia and cognitive decline following a stroke.

Current research reveals the importance of melatonin receptors, as the targeted cellular mechanism for melatonin-induced neuroprotection, as depicted in Figure 1. MT1 and MT2 are prominent all through the brain and thus, may serve as a potent therapeutic target in treatment of stroke and neurodegenerative diseases [67]. When melatonin receptors are impeded, the positive effects of melatonin in AD, specifically the repression of β-amyloid (Aβ) synthesis and fibril production, are eliminated. Interestingly, Parkinson disease patients demonstrate a depletion of MT1 and MT2 receptors in amygdala and substantia nigra pars compacta. In mice models of cerebral ischemia, melatonin spurs neurogenesis via MT2. Notably, MSCs’ neurogenic capabilities can be bolstered by melatonin, primarily due to the MT2 receptor [67]. In post hypoxic-ischemic (H-I) brain injury, MT1 receptors were substantially depleted in the brains of mouse pups. Melatonin alleviated ischemic injury by increasing MT1 receptors. As MT1 was blocked in the H-I mice, mortality rates escalated significantly. When luzindole, an antagonist of the melatonin receptor, was implemented, the therapeutic effects of melatonin were eliminated with respect to mitochondrial cell death. Moreover, melatonin’s rehabilitation of MT1 receptors generates its curative actions in ischemic injury [68]. In Wistar rats, MT1, MT2 and MT3 receptors ameliorated edema and utilizing estrogen, MT1 and MT2 safeguarded the BBB [69]. Evidently, MT2 activity, regulated by the cAMP-C/EBPα/miR-125b/GluN2A pathway, imparts neuroprotection in AD, specifically ameliorating dendritic damage caused by Aβ [70]. In addition, ramelteon, a melatonin receptor agonist utilized in insomnia treatment, was explored as a potential therapeutic target in cerebral ischemia. Ramelteon substantially improved function and hindered autophagy using AMPK/mTOR signaling in both acute and chronic stroke models. Importantly, MT antagonist, 4-P-PDOT, abolished the neuroprotective effects of ramelteon, indicating the therapeutic importance of the MT receptor [71]. Stem cells express MT1 and MT2 and therefore, investigating the involvement of melatonin in stem cell mechanisms is crucial. In a recent study, melatonin spurred differentiation of amniotic fluid (AF)- MSCs into dopaminergic neurons, potentially through the initiation of ERK and CaMKII signaling, regulated by melatonin receptors [72]. Moreover, targeting melatonin receptors in stem cells may further bolster their differentiative and neuroprotective effects. 

On account of melatonin’s plethora of neuroprotective features, stroke-induced dementia stands as a significant therapeutic target for melatonin-based treatment. In APP/PSI mice, a model of AD, long-term administration of melatonin ameliorated spatial learning and memory loss, curtailing Aβ accumulation and mitochondrial dysfunction [73]. Oxidative injury in the brain is a major factor inducing cognitive impairment and memory loss in AD. Melatonin suppressed the destructive effects of scopolamine-induced oxidative damage regulated by c-Jun N-terminal kinase (JNK) activation. Melatonin upregulated antioxidant proteins, decreased ROS and LPO and promoted Akt/ERK/CREB pathways, leading to heightened cell viability and proliferation. Melatonin also inhibited apoptosis and altogether improved synaptic impairment, memory loss, neuroinflammation and neurodegeneration [74]. In rats affected by intestinal I/R injury, melatonin alleviated cognitive impairment and decreased proinflammatory factors, such as tumor necrosis factor-α, IL-6 and interleukin-1β, along with attenuating oxidative stress in the brain, blood serum and intestinal tissue. Additionally, melatonin inhibited apoptosis and microglial hyperactivity in brain tissue [75]. Notably, melatonin restores hippocampal neurogenesis in mice treated with cuprizone by enhancing BDNF and increasing CREB phosphorylation [76]. Moreover, melatonin can ameliorate stroke-induced depletion of CREB and BDNF in hippocampal tissue, rehabilitating cognitive performance and memory. 

**Figure 2:**
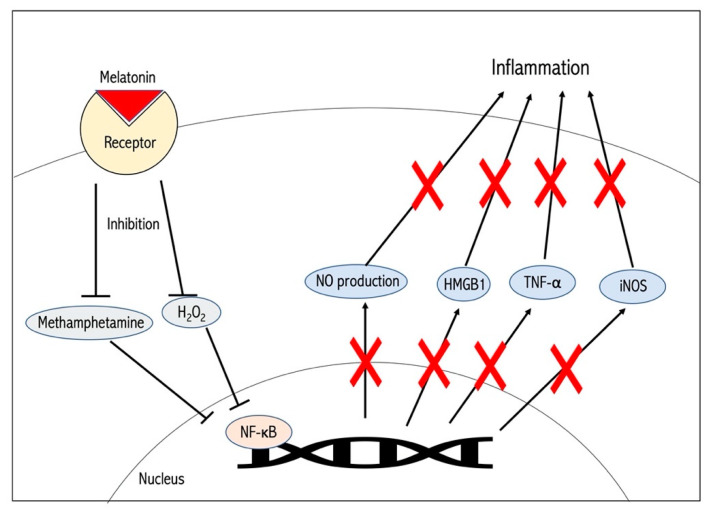
Melatonin’s Intracellular Signaling. Melatonin initiates a cellular pathway that inhibits Methamphetamine and hydrogen peroxide in the cell, resulting in the attenuation of inflammation and oxidative stress.

Regarding recent clinical trials, melatonin-based treatment has been utilized for stroke and neurodegenerative disease patients. 60 patients received an oral dosage of melatonin 3 days before and after carotid endarterectomy (CEA) surgery. Blood samples of the melatonin group demonstrated significant reduction of inflammatory factors, NF-κB, TNF-α, IL-6 and S100β and an elevation of antioxidant activity through Nrf2, SOD, CAT and GPx [77]. Furthermore, melatonin demonstrates significant therapeutic promise against ischemic brain damage on account of its antioxidant and anti-inflammatory capabilities [Figure 2]. In addition, multiple sclerosis (MS) pathology entails significant neuroinflammation and oxidative damage, leading to demyelination and neuronal injury. Patients with relapsing-remitting multiple sclerosis (RRMS) under Interferon β-1b (IFNβ-1b) treatment were given 25g/d of oral melatonin for six months. Compared to the placebo subjects, patients taking melatonin demonstrated a substantial reduction of pro-inflammatory cytokines, such as TNF-α, IL-6 and IL-10 and oxidative stress factors like nitric oxide catabolites and LPO [78]. Moreover, melatonin may display similar therapeutic benefits in clinical cases of stroke-induced oxidative damage and neuroinflammation. 

Nevertheless, further preclinical investigation into the clinical safety and efficacy of melatonin in post-stroke treatment is warranted. Experimental stroke in preclinical trials should examine the long-term effects to assess the safety of melatonin therapy, ensuring no adverse effects, such as behavioral side effects and neurological inconsistencies, are present. To further bolster the safety profile for clinical treatment, investigators should examine neuronal tissues and peripheral organs for potential toxic effects during various stages post-melatonin therapy. Stroke Treatment Academic Industry Roundtable (STAIR) and Stem Cell therapies as an Emerging Paradigm (STEP) propose guidelines to potentially transition previously discussed therapies to clinical trials [79,80]. Both STAIR and STEP recommend effective protocols developed by stroke research committees, outlining plans for conducting experimental and early clinical testing. These plans include tests that range from multiple lab tests to model tests and additional comorbidity factors, such as diabetes and aging. Successful implication of the guidelines modeled by stroke committees, STEP and STAIR, will effectively transition melatonin therapy from preclinical to clinical studies. Additionally, in vivo models have demonstrated the immunoenhancing effects of melatonin [81,82,83] and therefore, these mechanisms should be further investigated before initiating melatonin-based therapies in clinical settings. Indeed, recent clinical investigations have elucidated melatonin’s therapeutic potency; however, additional pre-clinical examination of the safety and efficacy of melatonin in the context of stroke is crucial for establishing proper clinical application. 

## 7. Conclusions

Stroke stands as a prominent agent of cognitive impairment and mortality worldwide and unfortunately has limited treatment options. Therefore, novel therapies like melatonin are crucial to attenuating stroke’s devastating effects. Melatonin acts as an antioxidant and free radical scavenger, ameliorating neuroinflammation and accelerating brain tissue restoration. Preclinical studies utilizing animal stroke models and clinical trials with stroke patients have been conducted to investigate melatonin’s therapeutic potency in the context of stroke. Experimental stroke models in vitro and in vivo have demonstrated melatonin’s neuroprotective capabilities, as melatonin reduces infarct size and enhances glial cell viability. Melatonin delivered exogenously displays substantial therapeutic effects but results fluctuate with changing melatonin levels. However, pineal gland grafts have the ability to provide long-term melatonin and therefore, may be useful for chronic treatment. Nonetheless, further examination of pineal gland transplantation efficacy is necessary. The mechanism behind melatonin-induced neuroprotection seems largely due to receptors MT1 and MT2, which can also be found in stem cells. Moreover, stem cell-based therapy for stroke and neurodegenerative disease may be bolstered by targeting the MT1 and MT2 receptors. Regarding stroke-related dementia, melatonin-based therapy may be a viable treatment option due to their neuroprotective properties, repressing inflammatory factors, oxidative stress and apoptotic pathways in stroke and AD models. In addition, melatonin enhances BDNF and CREB, alleviating cognitive impairment. Notably, recent clinical trials exploring the curative potential of melatonin in stroke and neurodegenerative diseases display encouraging results in alleviating brain injury. Nevertheless, additional preclinical studies examining the safety and efficacy of melatonin-based therapies in stroke and stroke-related dementia are warranted to ensure the treatments’ optimal clinical applications.

## Figures and Tables

**Table 1:** Melatonin Neuroprotection in Stroke. This table displays milestone discoveries highlighting melatonin as a potent therapeutic in stroke.

Model	Stroke Type	Significant Findings
In vivo	Permanent	Modulation of L-arginine metabolism via melatonin improves stroke outcomes. Post-middle cerebral artery occlusion administration of melatonin significantly reduced nitric oxide synthase activity, nitrite levels and cyclooxygenases, all of which contribute to stroke-induced inflammation. A decrease in infarct volume and rejuvenation of mitochondrial enzymatic activity was also observed [84].
In vivo	Transient	Activation of MT2 receptor via melatonin after transient middle cerebral artery injury and reperfusion significantly improved brain function and survival in mice. Free radical production and gp91(phox) cell infiltration were decreased, consequently preserving blood brain barrier function. Enhanced endogenous neurogenesis and expression of neurodevelopmental genes were also observed [85].
In vivo	Permanent	Melatonin administration ameliorates ischemic reperfusion injury via activating SIRT1 signaling and improving mitochondrial function. Neuroprotective effects were demonstrated in mice upon treatment with melatonin post-ischemia including reduced infarct volume, decreased edema and improved neurological scores. Activation of SIRT1 via melatonin upregulates anti-apoptotic factor Bcl2 and lowers expression of pro-apoptotic protein Bax indicating that melatonin possesses anti-apoptotic effects [86].
Clinical	Permanent	6-sulfatoximelatonin indicates post-stroke cognitive impairment in elderly patients. The presence of 6-sulfatoximelatonin, a metabolite of melatonin, was investigated in the urine of patients during the acute phase of stroke. Increased concentration of the metabolite was linked to large ischemic lesions and hippocampal volume. Patients with the highest concentrations of the metabolite presented with dysexecutive cognitive impairment [87].
In vivo	Permanent	Pre-treatment with melatonin before ischemia inhibits endoplasmic reticulum (ER) stress-dependent autophagy, shielding against cerebral ischemic/reperfusion (IR) injury. Pre-ischemic melatonin administration voided IR-associated ER stress autophagy and ameliorated autophagic flux. Melatonin also reduced edema, infarction size and apoptosis [88].
In vivo	Permanent	PI3K/Akt phosphorylation reduces apoptosis and mediates melatonin’s neuroprotective effects via PDK1 and PTEN at the Thr308 site. Melatonin’s neuroprotective effects were reversed in focal ischemia murine model by PI3K/Akt inhibition, specifically reduction in infarct volume, indicating P13K/Akt are involved in melatonin’s ameliorative effects. The PI3K/Akt pathway decreased p53 phosphorylation consequently reducing apoptosis [89].
In vivo	Permanent	Melatonin alleviates symptoms associated with secondary brain injury (SBI) following intracerebral hemorrhage (ICH) in rats. Administration of melatonin significantly decreased concentration of inflammatory, DNA damage, oxidative stress, blood brain barrier integrity and apoptosis markers. Mitochondrial function was maintained via decreasing membrane permeability and transition pore opening. Melatonin also ameliorated brain edema, improved behavior and upregulated antioxidant indicator expression [90].
In vivo	Permanent	Melatonin displays favorable outcomes when administered to aged rats pre-ischemia and post-ischemia. Rats subject to MCAO were treated with melatonin 24 h before pre-ischemia and data indicated reduced levels of tumor necrosis factor-α, Bcl-2-associated death promoter, interleukin-1β, Bcl-2-associated X protein glial fibrillary acidic protein in the hippocampus and cortex. Augmented levels of sirtuin 1 and B-cell lymphoma were observed in the hippocampal region. Post-ischemic treatment provided similar effects; however these were not as effective [91].
Clinical	Permanent	Peroxidation status, mortality and antioxidant status are linked to melatonin concentration in patients with middle cerebral artery infarction. Non-survivors presented with significantly increased antioxidant capacity, malondialdehyde and serum melatonin levels when compared to survivors. A positive associated was observed between serum melatonin levels with total antioxidant capacity and malondialdehyde concentration [92].
In vivo	Transient	Long term melatonin treatment post-transient global cerebral ischemia (tGCI) improves outcomes via activation of ERK1/2 signaling. Treatment ameliorated cognitive impairment and expanded myelin basic protein immunoreactivity and levels of Rip-immunoreactive oligodendrocytes. ERK1/2 and pERK1/2 activity was increased in oligodendrocytes. Glutamatergic synapse activity was also augmented through long term-melatonin treatment post tGCI [93].
In vivo	Permanent	The shift of microglia from pro-inflammatory to anti-inflammatory polarity in STAT3-dependent manner via melatonin partially improves brain function after distal middle cerebral artery occlusion. Reduced infarct volume, improved brain function, inhibition of pro-inflammatory responses was observed after melatonin administration. Melatonin increased phosphorylated STAT3 expression in BV2 cells [58].
In vitro	Permanent	Modulation of microglial action via melatonin ameliorates reperfusion phase-induced secondary injury after stroke. In vitro, melatonin treatment early in the reperfusion phase improved outcomes. GFAP, Iba1, active caspase-3 all decreased upon administration while NeuN increased. BDNF, HSPA1A and MAP2 were seen at augmented levels while VEGF mRNA was decreased. TREM2/iNOS ratio increased indicating protective forms of microglia [61].

## References

[1] Tan D., Manchester L.C., Reiter R.J., Qi W., Hanes M.A., Farley N.J. (1999). High physiological levels of melatonin in the bile of mammals. Life Sci..

[2] Skinner D.C., Malpaux B. (1999). High melatonin concentrations in third ventricular cerebrospinal fluid are not due to Galen vein blood recirculating through the choroid plexus. Endocrinology.

[3] Yu H.S., Yee R.W., Howes K.A., Reiter R.J. (1990). Diurnal rhythms of immunoreactive melatonin in the aqueous humor and serum of male pigmented rabbits. Neurosci. Lett..

[4] Reiter R.J., Tan D.X., Fuentes-Broto L. (2010). Melatonin: A multitasking molecule. Prog. Brain Res..

[5] Reiter R.J. (1998). Oxidative damage in the central nervous system: Protection by melatonin. Prog. Neurobiol..

[6] Cutler R.G. (1995). Oxidative stress: Its potential relevance to human disease and longevity determinants. AGE.

[7] Leker R.R., Teichner A., Lavie G., Shohami E., Lamensdorf I., Ovadia H. (2002). The nitroxide antioxidant tempol is cerebroprotective against focal cerebral ischemia in spontaneously hypertensive rats. Exp. Neurol..

[8] Cuzzocrea S., Costantino G., Caputi A.P. (1998). Protective effect of melatonin on cellular energy depletion mediated by peroxynitrite and poly (ADP-ribose) synthetase activation in a non-septic shock model induced by zymosan in the rat. J. Pineal Res..

[9] Cagnoli C.M., Atabay C., Kharlamova E., Manev H. (1995). Melatonin protects neurons from singlet oxygen-induced apoptosis. J. Pineal Res..

[10] Kilic E., Ozdemir Y.G., Bolay H., Kelestimur H., Dalkara T. (1999). Pinealectomy aggravates and melatonin administration attenuates brain damage in focal ischemia. J. Cereb. Blood Flow Metab..

[11] Lee E.J., Lee M.Y., Chen H.Y., Hsu Y.S., Wu T.S., Chen S.T., Chang G.L. (2005). Melatonin attenuates gray and white matter damage in a mouse model of transient focal cerebral ischemia. J. Pineal Res..

[12] Pei Z., Pang S.F., Cheung R.T. (2002). Pretreatment with melatonin reduces volume of cerebral infarction in a rat middle cerebral artery occlusion stroke model. J. Pineal Res..

[13] Kaneko Y., Hayashi T., Yu S., Tajiri N., Solomita M.A., Chheda S.H., Weinbren N.L., Parolini O., Borlongan C.V. (2011). Human amniotic epithelial cells express melatonin receptor MT1 but not melatonin receptor MT2: A new perspective to neuroprotection. J. Pineal Res..

[14] Lee M.Y., Kuan Y.H., Chen H.Y., Chen T.Y., Chen S.T., Huang C.C., Yang I.P., Hsu Y.S., Wu T.S., Lee E.J. (2007). Intravenous administration of melatonin reduces the intracerebral cellular inflammatory response following transient focal cerebral ischemia in rats. J. Pineal Res..

[15] Shinozuka K., Staples M., Borlongan C.V. (2013). Melatonin-based therapeutics for neuroprotection in stroke. Int. J. Mol. Sci..

[16] Wang Y., Lin S.Z., Chiou A.L., Williams L.R., Hoffer B.J. (1997). Glial cell line-derived neurotrophic factor protects against ischemia-induced injury in the cerebral cortex. J. Neurosci..

[17] Luscher C., Malenka R.C., Nicoll R.A. (1998). Monitoring glutamate release during LTP with glial transporter currents. Neuron.

[18] Redmond D.E. (2002). Cellular replacement therapy for Parkinson’s disease—Where we are today?. Neuroscientist.

[19] Kondziolka D., Wechsler L., Goldstein S., Meltzer C., Thulborn K.R., Gebel J., Jannetta P., DeCesare S., Elder E.M., McGrogan M. (2000). Transplantation of cultured human neuronal cells for patients with stroke. Neurology.

[20] Kondoh T., Uneyama H., Nishino H., Torii K. (2002). Melatonin reduces cerebral edema formation caused by transient forebrain ischemia in rats. Life Sci..

[21] Borlongan C.V., Sumaya I., Moss D., Kumazaki M., Sakurai T., Hida H., Nishino H. (2003). Melatonin-secreting pineal gland: A novel tissue source for neural transplantation therapy in stroke. Cell Transplant..

[22] Gupta Y.K., Chaudhary G., Sinha K. (2002). Enhanced protection by melatonin and meloxicam combination in a middle cerebral artery occlusion model of acute ischemic stroke in rat. Can. J. Physiol. Pharmacol..

[23] Lee S., Shin J., Hong Y., Lee M., Kim K., Lee S.R., Chang K.T., Hong Y. (2012). Beneficial effects of melatonin on stroke-induced muscle atrophy in focal cerebral ischemic rats. Lab. Anim. Res..

[24] Kahan B.D., Gholerial R. (1994). Immunosuppressive agents. Surg. Clin. N. Am..

[25] Ben-Nathan D., Maestroni G.J.M., Lustig S., Conti S. (1995). Protective effects of melatonin in mice infected with encephalitis virus. Arch. Virol..

[26] Ben-Nathan D., Maestroni G.J.M., Conti A., Maestroni G.J.M., Conti A., Reiter R.J. (1997). The protective effect of melatonin in viral and bacterial infections. Therapeutic Potential of Melatonin.

[27] Maestroni G.J. (1993). The immunoendocrine role of melatonin. J. Pineal Res..

[28] Borlongan C.V., Sanberg P.R. (2002). Neural transplantation for treatment of Parkinson’s disease. Drug Discov. Today.

[29] Sharma R., McMillan C.R., Niles L.P. (2007). Neural stem cell transplantation and melatonin treatment in a 6-hydroxydopamine model of Parkinson’s disease. J. Pineal Res..

[30] Niles L.P., Armstrong K.J., Castro L.M., Dao C.V., Sharma R., McMillan C.R., Doering L.C., Kirkham D.L. (2004). Neural stem cells express melatonin receptors and neurotrophic factors: Colocalization of the MT1 receptor with neuronal and glial markers. BMC Neurosci..

[31] Beni S.M., Kohen R., Reiter R.J., Tan D.X., Shohami E. (2004). Melatonin-induced neuroprotection after closed head injury is associated with increased brain antioxidants and attenuated late-phase activation of NF-κB and AP-1. FASEB J..

[32] Moriya T., Horie N., Mitome M., Shinohara K. (2007). Melatonin influences the proliferative and differentiative activity of neural stem cells. J. Pineal Res..

[33] Lekic T., Hartman R., Rojas H., Manaenko A., Chen W., Ayer R., Tang J., Zhang J.H. (2010). Protective effect of melatonin upon neuropathology, striatal function and memory ability after intracerebral hemorrhage in rats. J. Neurotrauma.

[34] Lin H.W., Lee E.J. (2009). Effects of melatonin in experimental stroke models in acute, sub-acute and chronic stages. Neuropsychiatr. Dis. Treat..

[35] Kong X., Li X., Cai Z., Yang N., Liu Y., Shu J., Pan L., Zuo P. (2008). Melatonin regulates the viability and differentiation of rat midbrain neural stem cells. Cell. Mol. Neurobiol..

[36] Kilic E., Kilic U., Bacigaluppi M., Guo A., Abdallah N.B., Wolfer D.P., Reiter R.J., Hermann D.M., Bassetti C.L. (2008). Delayed melatonin administration promotes neuronal survival, neurogenesis and motor recovery and attenuates hyperactivity and anxiety after mild focal cerebral ischemia in mice. J. Pineal Res..

[37] Ramirez-Rodriguez G., Klempin F., Babu H., Benitez-King G., Kempermann G. (2009). Melatonin modulates cell survival of new neurons in the hippocampus of adult mice. Neuropsychopharmacology.

[38] Xu S.C., He M.D., Zhong M., Zhang Y.W., Wang Y., Yang J., Yu Z.P., Zhou Z. (2010). Melatonin protects against Nickel-induced neurotoxicity in vitro by reducing oxidative stress and maintaining mitochondrial function. J. Pineal Res..

[39] Romeu L.R., da Motta E.L., Maganhin C.C., Oshima C.T., Fonseca M.C., Barrueco K.F., Simoes R.S., Pellegrino R., Baracat E.C., Soares-Junior J.M. (2011). Effects of melatonin on histomorphology and on the expression of steroid receptors, VEGF and PCNA in ovaries of pinealectomized female rats. Fertil. Steril..

[40] Imbesi M., Uz T., Manev H. (2008). Role of melatonin receptors in the effects of melatonin on BDNF and neuroprotection in mouse cerebellar neurons. J. Neural Transm..

[41] Lee C.H., Yoo K.Y., Choi J.H., Park O.K., Hwang I.K., Kwon Y.G., Kim Y.M., Won M.H. (2010). Melatonin’s protective action against ischemic neuronal damage is associated with up-regulation of the MT2 melatonin receptor. J. Neurosci. Res..

[42] Mao L.L., Cheng Q., Guardiola-Lemaitre B., Schuster-Klein C., Dong C., Lai L., Hill S.M. (2010). In vitro and in vivo antitumor activity of melatonin receptor agonists. J. Pineal Res..

[43] Mor M., Rivara S., Pala D., Bedini A., Spadoni G., Tarzia G. (2010). Recent advances in the development of melatonin MT1 and MT2 receptor agonists. Expert Opin. Ther. Pat..

[44] Hardeland R., Poeggeler B., Srinivasan V., Trakht I., Pandi-Perumal S.R., Cardinali D.P. (2008). Melatonergic drugs in clinical practice. Arzneimittelforschung.

[45] Simpson D., Curran M.P. (2008). Ramelteon—A review of its use in insomnia. Drugs.

[46] Kalaria R.N. (2012). Risk factors and neurodegenerative mechanisms in stroke related dementia. Panminerva Med..

[47] Zhang X., Bi X. (2020). Post-Stroke Cognitive Impairment: A Review Focusing on Molecular Biomarkers. J. Mol. Neurosci..

[48] Zhao Y., Xiao M., He W., Cai Z. (2015). Minocycline upregulates cyclic AMP response element binding protein and brain-derived neurotrophic factor in the hippocampus of cerebral ischemia rats and improves behavioral deficits. Neuropsychiatr. Dis. Treat..

[49] Goulay R., Romo L.M., Hol E.M., Dijkhuizen R.M. (2020). From Stroke to Dementia: A Comprehensive Review Exposing Tight Interactions between Stroke and Amyloid-β Formation. Transl. Stroke Res..

[50] Suzuki A., Fukushima H., Mukawa T., Toyoda H., Wu L.J., Zhao M.G., Xu H., Shang Y., Endoh K., Iwamoto T. (2011). Upregulation of CREB-mediated transcription enhances both short- and long-term memory. J. Neurosci..

[51] Gumuslu E., Mutlu O., Sunnetci D., Ulak G., Celikyurt I.K., Cine N., Akar F., Savli H., Erden F. (2014). The antidepressant agomelatine improves memory deterioration and upregulates CREB and BDNF gene expression levels in unpredictable chronic mild stress (UCMS)-exposed mice. Drug Target Insights.

[52] Yang C.L., Guo H., Zhou H., Suo D.Q., Li W.J., Zhou Y., Zhao Y., Yang W.S., Jin X. (2015). Chronic oleoylethanolamide treatment improves spatial cognitive deficits through enhancing hippocampal neurogenesis after transient focal cerebral ischemia. Biochem. Pharmacol..

[53] Chen L., Qiu R., Li L., He D., Lv H., Wu X., Gu N. (2014). The role of exogenous neural stem cells transplantation in cerebral ischemic stroke. J. Biomed. Nanotechnol..

[54] Jeong H.C., Kim M.S., Lim Y.J., Ryu C.H., Jun J.A., Jeun S.S. (2014). Mesenchymal stem cells expressing brain-derived neurotrophic factor enhance endogenous neurogenesis in an ischemic stroke model. Biomed. Res. Int..

[55] Tan S., Zhi P.K., Luo Z.K., Shi J. (2015). Severe instead of mild hyperglycemia inhibits neurogenesis in the subventricular zone of adult rats after transient focal cerebral ischemia. Neuroscience.

[56] Kaneko N., Kako E., Sawamoto K. (2013). Enhancement of ventricular-subventricular zone-derived neurogenesis and oligodendrogenesis by erythropoietin and its derivatives. Front. Cell. Neurosci..

[57] Tan H.Y., Ng K.Y., Koh R.Y., Chye S.M. (2020). Pharmacological Effects of Melatonin as Neuroprotectant in Rodent Model: A Review on the Current Biological Evidence. Cell. Mol. Neurobiol..

[58] Liu Z.J., Ran Y.Y., Qie S.Y., Wei-Jun G., Fu-Hai G., Zi-Tong D., Jia-Ning X. (2019). Melatonin protects against ischemic stroke by modulating microglia/macrophage polarization toward anti-inflammatory phenotype through STAT3 pathway. CNS Neurosci. Ther..

[59] Zhi S.M., Fang G.X., Xie X.M., Liu L.H., Yan J., Liu D.B., Yu H.Y. (2020). Melatonin reduces OGD/R-induced neuron injury by regulating redox/inflammation/apoptosis signaling. Eur. Rev. Med. Pharmacol. Sci..

[60] Motallebzade E., Tameh A.A., Zavareh S.A.T., Farhood B., Aliasgharzedeh A., Mohseni M. (2020). Neuroprotective effect of melatonin on radiation-induced oxidative stress and apoptosis in the brainstem of rats. J. Cell. Physiol..

[61] Azedi F., Mehrpour M., Talebi S., Zendedel A., Kazemnejad S., Mousavizadeh K., Beyer C., Zarnani A.H., Joghataei M.T. (2019). Melatonin regulates neuroinflammation ischemic stroke damage through interactions with microglia in reperfusion phase. Brain Res..

[62] Michalska P., Buendia I., Duarte P., Mendivil C.F. (2020). Melatonin-sulforaphane hybrid ITH12674 attenuates glial response in vivo by blocking LPS binding to MD2 and receptor oligomerization. Pharmacol. Res..

[63] Wei N., Pu Y., Yang Z., Pan Y., Liu L. (2019). Therapeutic effects of melatonin on cerebral ischemia reperfusion injury: Role of Yap-OPA1 signaling pathway and mitochondrial fusion. Biomed. Pharmacother..

[64] Jana T., Tzveta S., Zlatina N., Natasha I., Dimitrinka A., Milena A., Katerina G. (2020). Effect of endurance training on diurnal rhythms of superoxide dismutase activity, glutathione and lipid peroxidation in plasma of pinealectomized rats. Neurosci. Lett..

[65] Doğanlar Z.B., Güçlü H., Öztopuz Ö., Türkön H., Dogan A., Uzun M., Doğanlar O. (2019). The Role of Melatonin in Oxidative Stress, DNA Damage, Apoptosis and Angiogenesis in Fetal Eye under Preeclampsia and Melatonin Deficiency Stress. Curr. Eye Res..

[66] Ramezani M., Komaki A., Hashemi-Firouzi N., Mortezaee K., Faraji N., Golipoor Z. (2020). Therapeutic effects of melatonin-treated bone marrow mesenchymal stem cells (BMSC) in a rat model of Alzheimer’s disease. J. Chem. Neuroanat..

[67] Wongprayoon P., Govitrapong P. (2020). Melatonin receptor as a drug target for neuroprotection. Curr. Mol. Pharmacol..

[68] Sinha B., Wu Q., Li W., Tu Y., Sirianni A.C., Chen Y., Jiang J., Zhang X., Chen W., Zhou S. (2018). Protection of melatonin in experimental models of newborn hypoxic-ischemic brain injury through MT1 receptor. J. Pineal Res..

[69] Shahrokhi N., Khaksari M., AsadiKaram G., Soltani Z., Shahrokhi N. (2018). Role of melatonin receptors in the effect of estrogen on brain edema, intracranial pressure and expression of aquaporin 4 after traumatic brain injury. Iran J. Basic Med. Sci..

[70] Tang H., Ma M., Wu Y., Deng M.F., Hu F., Almansoub H.A., Huang H.Z., Wang D.Q., Zhou L.T., Wei N. (2019). Activation of MT2 receptor ameliorates dendritic abnormalities in Alzheimer’s disease via C/EBPα/miR-125b pathway. Aging Cell.

[71] Wu X.L., Lu S.S., Liu M.R., Tang W.D., Chen J.Z., Zheng Y.R., Ahsan A., Cao M., Jiang L., Hu W.W. (2020). Melatonin receptor agonist ramelteon attenuates mouse acute and chronic ischemic brain injury. Acta Pharmacol. Sin..

[72] Phonchai R., Phermthai T., Kitiyanant N., Suwanjang W., Kotchabhakdi N., Chetsawang B. (2019). Potential effects and molecular mechanisms of melatonin on the dopaminergic neuronal differentiation of human amniotic fluid mesenchymal stem cells. Neurochem. Int..

[73] Sun C., Qiu X., Wang Y., Liu J., Li Q., Jiang H., Li S., Song C. (2020). Long-term oral melatonin alleviates memory deficits, reduces amyloid-β deposition associated with downregulation of BACE1 and mitophagy in APP/PS1 transgenic mice. Neurosci. Lett..

[74] Muhammad T., Ali T., Ikram M., Khan A., Alam S.I., Kim M.O. (2019). Melatonin Rescue Oxidative Stress-Mediated Neuroinflammation/Neurodegeneration and Memory Impairment in Scopolamine-Induced Amnesia Mice Model. J. Neuroimmune Pharmacol..

[75] Yang B., Zhang L.Y., Chen Y., Bai Y.P., Jia J., Feng J.G., Liu K.X., Zhou J. (2020). Melatonin alleviates intestinal injury, neuroinflammation and cognitive dysfunction caused by intestinal ischemia/reperfusion. Int. Immunopharmacol..

[76] Kim W., Hahn K.R., Jung H.Y., Kwon H.J., Nam S.M., Kim J.W., Park J.H., Yoo D.Y., Kim D.W., Won M.H. (2019). Melatonin ameliorates cuprizone-induced reduction of hippocampal neurogenesis, brain-derived neurotrophic factor and phosphorylation of cyclic AMP response element-binding protein in the mouse dentate gyrus. Brain Behav..

[77] Zhao Z., Lu C., Li T., Wang W., Ye W., Zeng R., Ni L., Lai Z., Wang X., Liu C. (2018). The protective effect of melatonin on brain ischemia and reperfusion in rats and humans: In vivo assessment and a randomized controlled trial. J. Pineal Res..

[78] Sánchez-López A.L., Ortiz G.G., Pacheco-Moises F.P., Mireles-Ramírez M.A., Bitzer-Quintero O.K., Delgado-Lara D.L., Ramírez-Jirano L.J., Velázquez-Brizuela I.E. (2018). Efficacy of Melatonin on Serum Pro-inflammatory Cytokines and Oxidative Stress Markers in Relapsing Remitting Multiple Sclerosis. Arch. Med. Res..

[79] Albers G.W., Goldstein L.B., Hess D.C., Wechsler L.R., Fuire K.L., Gorelick P.B., Hurn P., Liebskind D.S., Nogueira R.G., Saver J.L. (2011). Stroke Treatment Academic Industry Roundtable (STAIR) recommendations for maximizing the use of intravenous thrombolytics and expanding treatment options with intra-arterial and neuroprotective therapies. Stroke.

[80] STEPS Participants (2009). Stem Cell Therapies as an Emerging Paradigm in Stroke Participants. Stem Cell Therapies as an Emerging Paradigm in Stroke (STEPS): Bridging basic and clinical science for cellular and neurogenic factor therapy in treating stroke. Stroke.

[81] Currier N.L., Sun L.Z., Miller S.C. (2000). Exogenous melatonin: Quantitative enhancement in vivo of cells mediating non-specific immunity. J. Neuroimmunol..

[82] Carillo-Vico A., Guerrero J.M., Lardone P.J., Reiter R.J. (2005). A review of the multiple actions of melatonin on the immune system. Endocrine.

[83] Giordano M., Palermo M.S. (1991). Melatonin-induced enhancement of antibody-dependent cellular cytotoxicity. J. Pineal Res..

[84] Nair S.M., Rahman R.M., Clarkson A.N., Sutherland B.A., Taurin S., Sammut I.A., Appleton I. (2011). Melatonin treatment following stroke induction modulates L-arginine metabolism. J. Pineal Res..

[85] Chern C.M., Liao J.F., Wang Y.H., Shen Y.C. (2012). Melatonin ameliorates neural function by promoting endogenous neurogenesis through the MT2 melatonin receptor in ischemic-stroke mice. Free Radic. Biol. Med..

[86] Yang Y., Jiang S., Dong Y., Fan C., Zhao L., Yang X., Li J., Di S., Yue L., Liang G. (2015). Melatonin prevents cell death and mitochondrial dysfunction via a SIRT1-dependent mechanism during ischemic-stroke in mice. J. Pineal Res..

[87] Kulesh A.A., Drobakha V.E., Shestakov V.V. (2016). The role of melatonin in the development of post-stroke cognitive impairment in elderly patients in comparison with middle-aged patients. Adv. Gerontol..

[88] Feng D., Wang B., Wang L., Abraham N., Tao K., Huang L., Shi W., Dong Y., Qu Y. (2017). Pre-ischemia melatonin treatment alleviated acute neuronal injury after ischemic stroke by inhibiting endoplasmic reticulum stress-dependent autophagy via PERK and IRE1 signalings. J. Pineal Res..

[89] Kilic U., Caglayan A.B., Beker M.C., Gunal M.Y., Caglayan B., Yalcin E., Kelestemur T., Gundogdu R.Z., Yulug B., Yılmaz B. (2017). Particular phosphorylation of PI3K/Akt on Thr308 via PDK-1 and PTEN mediates melatonin’s neuroprotective activity after focal cerebral ischemia in mice. Redox Biol..

[90] Wang Z., Zhou F., Dou Y., Tian X., Liu C., Li H., Shen H., Chen G. (2018). Melatonin Alleviates Intracerebral Hemorrhage-Induced Secondary Brain Injury in Rats via Suppressing Apoptosis, Inflammation, Oxidative Stress, DNA Damage and Mitochondria Injury. Transl. Stroke Res..

[91] Rancan L., Paredes S.D., García C., González P., Rodríguez-Bobada C., Calvo-Soto M., Hyacinthe B., Vara E., Tresguerres J.A.F. (2018). Comparison of the Effect of Melatonin Treatment before and after Brain Ischemic Injury in the Inflammatory and Apoptotic Response in Aged Rats. Int. J. Mol. Sci..

[92] Lorente L., Martín M.M., Abreu-González P., Pérez-Cejas A., Ramos L., Argueso M., Solé-Violán J., Cáceres J.J., Jiménez A., García-Marín V. (2018). Serum melatonin levels are associated with mortality in patients with malignant middle cerebral artery infarction. J. Int. Med. Res..

[93] Chen B.H., Park J.H., Lee Y.L., Kang I.J., Kim D.W., Hwang I.K., Lee C.H., Yan B.C., Kim Y.M., Lee T.K. (2018). Melatonin improves vascular cognitive impairment induced by ischemic stroke by remyelination via activation of ERK1/2 signaling and restoration of glutamatergic synapses in the gerbil hippocampus. Biomed. Pharmacother..

